# Inflammatory drivers of carotid atherosclerosis: signaling pathways, immune cells, and plaque stability

**DOI:** 10.3389/fcvm.2026.1919835

**Published:** 2026-09-16

**Authors:** Yingwei Li, Yanfei Yang, Yuhang Liu, Jianjun Jiang

**Affiliations:** 1 Department of Vascular Surgery, Qilu Hospital of Shandong University, Jinan, Shandong Province, China; 2 Department of Vascular Surgery, Peking Union Medical College Hospital, Chinese Academy of Medical Sciences & Peking Union Medical College, Beijing, China; 3 Dalian Medical University, Dalian, Liaoning Province, China

**Keywords:** carotid atherosclerosis, immune cell polarization, inflammatory signaling pathways, NLRP3 inflammasome, plaque stability

## Abstract

**Background:**

Carotid atherosclerosis (CAS; herein referring to carotid atherosclerotic disease, to be distinguished from carotid artery stenting, which shares the same abbreviation) is a primary driver of ischemic stroke, with inflammation playing a central role in its pathogenesis from plaque initiation to rupture. However, the complex regulatory network governing this inflammatory process remains to be fully elucidated.

**Purpose:**

This review comprehensively reviews the molecular and cellular inflammatory mechanisms underlying CAS, focusing on key signaling pathways, inflammatory mediators, and immune cell populations, to identify potential targets for therapeutic intervention. As a narrative review, it does not follow a formal systematic-search protocol.

**Key findings:**

We delineate the hierarchical activation of core signaling cascades—including the central NF-κB pathway, the MAPK and JAK/STAT modules, and the NLRP3 inflammasome—which collectively orchestrate a pro-inflammatory milieu. This network drives the expression of critical mediators (e.g., TNF-α, IL-1β, MMPs) and sculpts the behavior of lesional immune cells. Specifically, the imbalance between pro-inflammatory M1 macrophages together with Th1/Th17 effector T cells, vs. anti-inflammatory M2 macrophages and Tregs emerges as a key determinant of plaque stability.

**Conclusion:**

and Implications: The pathogenesis of CAS is governed by an intricate, multi-level inflammatory network. A detailed understanding of this network, particularly the crosstalk between signaling pathways and immune cell plasticity, provides a rationale for novel precision therapies. Targeting specific nodes within this network, rather than broad immunosuppression, holds promise for stabilizing high-risk plaques and reducing the burden of cerebrovascular events.

## 1 Introduction

Carotid atherosclerosis (CAS) is a major causative factor of cerebrovascular disease and a significant contributor to ischemic stroke, which has become the second leading cause of death after heart disease in China (1). Carotid stenosis ≥50% not only markedly increases the incidence of local cerebral ischemia but is also closely associated with stroke recurrence, making it a priority for prevention and treatment (1). Moreover, with the accelerating trend of population aging, the prevalence of CAS is rising annually, imposing a heavy burden on healthcare systems (2).

Inflammation is now recognized as the core mechanism driving the development and progression of CAS. The inflammatory process involves multiple cell types—including endothelial cells, mononuclear macrophages, smooth muscle cells, and lymphocytes—which interact through complex molecular signaling networks that drive plaque formation and progression (3). Notably, immune cells and their secreted inflammatory mediators play a pivotal role in plaque instability and rupture, directly influencing severe complications such as stroke (3, 4). For instance, macrophage activation not only promotes the release of inflammatory factors but also exacerbates local tissue damage through phagocytosis and inflammatory cascades, leading to disruption of vascular wall structure and increased plaque vulnerability (5, 6). Furthermore, the differentiation and polarization states of T cells are closely associated with atherosclerotic progression, with certain T cell phenotypes intensifying inflammatory responses while others may exert regulatory effects (5, 7).

Recent advances in molecular biology and immunology have begun to elucidate the specific inflammatory mechanisms underlying CAS. Multiple inflammatory signaling pathways, such as the NLRP3 inflammasome, the NF-κB pathway, and epigenetic regulatory factors, participate in plaque formation and the regulation of plaque stability (7, 8). Concurrently, non-coding RNA molecules, including microRNAs (miRNAs) and long non-coding RNAs (lncRNAs), have been found to regulate inflammatory gene expression, influence macrophage phenotype and function, and thereby participate in lesion regulation (9–11). Moreover, the response of vascular endothelial cells to hemodynamic forces influences atherosclerosis progression by modulating inflammatory states. Stable laminar shear stress induces the expression of anti-inflammatory molecules, protecting endothelial function, whereas disturbed flow promotes inflammation and plaque formation (12, 13).

These advances not only deepen our understanding of the inflammatory mechanisms underlying CAS but also offer new insights for precision medicine and targeted therapies. Modulating specific inflammatory mediators or signaling pathways to suppress pro-inflammatory cell activity or enhance anti-inflammatory responses holds promise for improving plaque stability and reducing stroke risk (7, 14). Concurrently, molecular subtyping and genetic analysis may aid in identifying high-risk patients, enabling personalized risk assessment and intervention strategies. Furthermore, interventional techniques such as carotid endarterectomy and stenting, combined with pharmacotherapy, have become essential clinical tools; however, their safety and timing require optimization based on deeper insights into inflammatory mechanisms (15, 16).

In summary, inflammation plays a central role in the progression of CAS, a key pathogenic driver of cerebrovascular disease (17). Recent advances have revealed a multi-level, multi-pathway regulatory network, providing theoretical foundations and practical guidance for early diagnosis, risk assessment, and precision treatment. This review comprehensively summarizes the latest research on inflammatory mechanisms in CAS, focusing on the roles of inflammatory mediators, immune cells, and their associated signaling pathways. We further explore their impact on plaque formation and stability, aiming to provide scientific evidence for clinical prevention and treatment (Figure 1).

**Figure 1:**
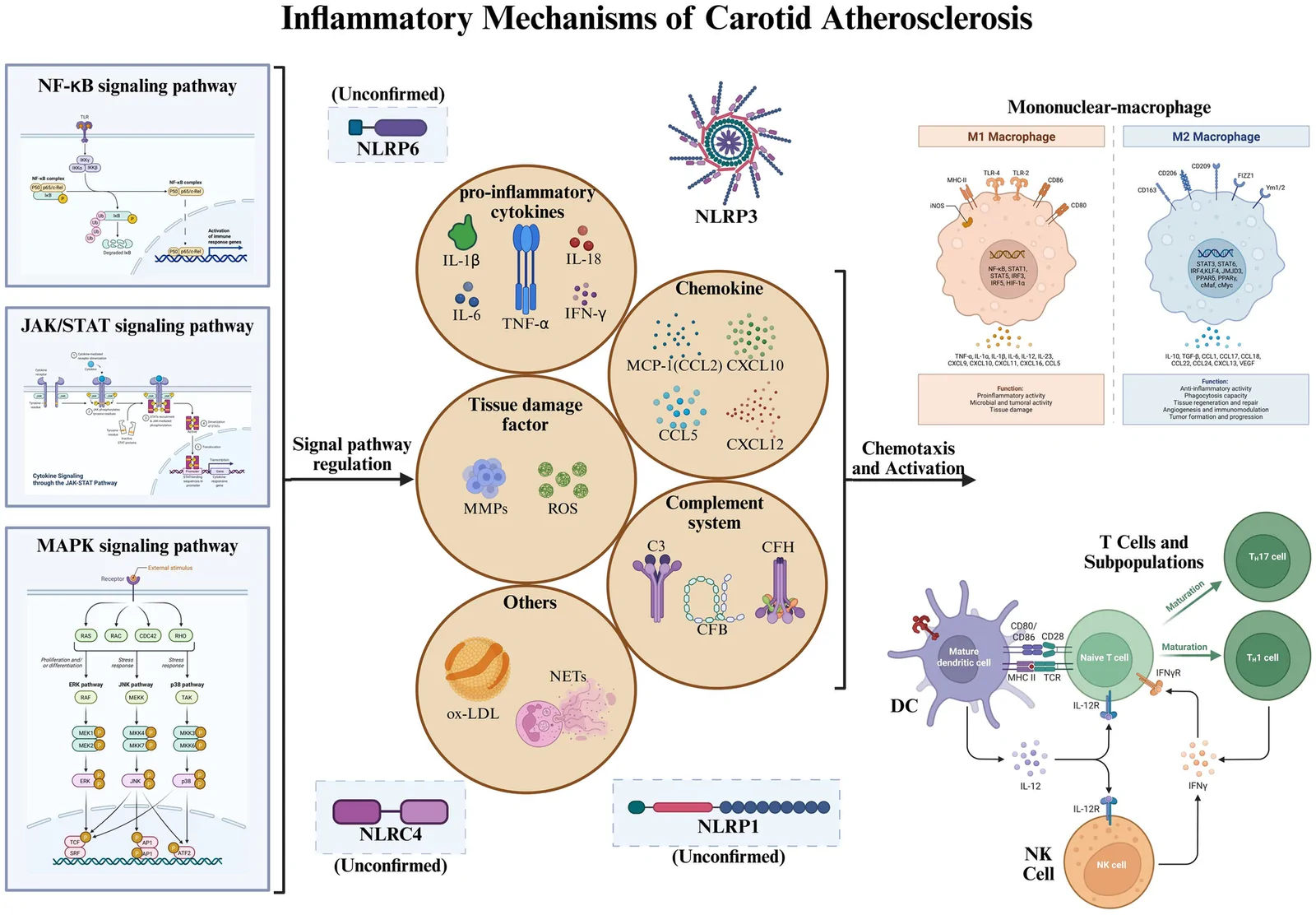
Overview of the inflammatory network driving carotid atherosclerosis (CAS). The schematic integrates the core signaling hubs (NF-κB, MAPK, JAK/STAT) with the NLRP3 inflammasome and the principal immune cell populations (macrophages, T cell subsets, and other innate cells) within the carotid plaque microenvironment. Arrows indicate the direction of regulation (activation, solid; inhibition, blunt-ended). Inflammasomes without an established role in CAS (NLRP1, NLRP6, NLRC4) are shown in dashed outline to denote that their plaque-specific function remains unconfirmed, per reviewer comment. First cited in the Introduction. Figure created with BioRender (bio-render.com).

## 2 Inflammatory signaling, mediators, and immune cells in carotid atherosclerosis

### 2.1 Activation and regulation of the NF-κB signaling pathway

In the inflammatory response to CAS, the nuclear factor κB (NF-κB) signaling pathway serves as a central regulator of multiple inflammatory genes (18). Studies indicate that RXRα modulates autophagy, thereby influencing p62/SQSTM1-mediated NF-κB activation (19, 20), and that functional inhibition of RXRα significantly attenuates plaque progression and inflammatory responses (19).

The canonical NF-κB pathway is triggered by various extracellular stimuli, including TNF-α, IL-1β, and LPS, which bind to their respective receptors such as TNFR1, IL-1R, and TLR4 (21–23). This binding induces receptor conformational changes, recruiting intracellular adaptor proteins (e.g., TRADD, MyD88, IRAK1) and ubiquitin ligases (e.g., TRAF2/6, cIAP1/2) to form a multiprotein complex on the inner leaflet of the cell membrane (24). Downstream, K63-linked ubiquitin chains recruit and activate the IKK complex (IKKα/IKKβ/NEMO); IKKβ phosphorylates IκBα, targeting it for K48-linked ubiquitination and proteasomal degradation, freeing p50/p65 to translocate to the nucleus and drive transcription of pro-inflammatory genes (25, 26). Signal termination is achieved by resynthesized IκBα and by the deubiquitinase A20 (27, 28). The detailed stepwise biochemistry is illustrated in Figure 2.

**Figure 2:**
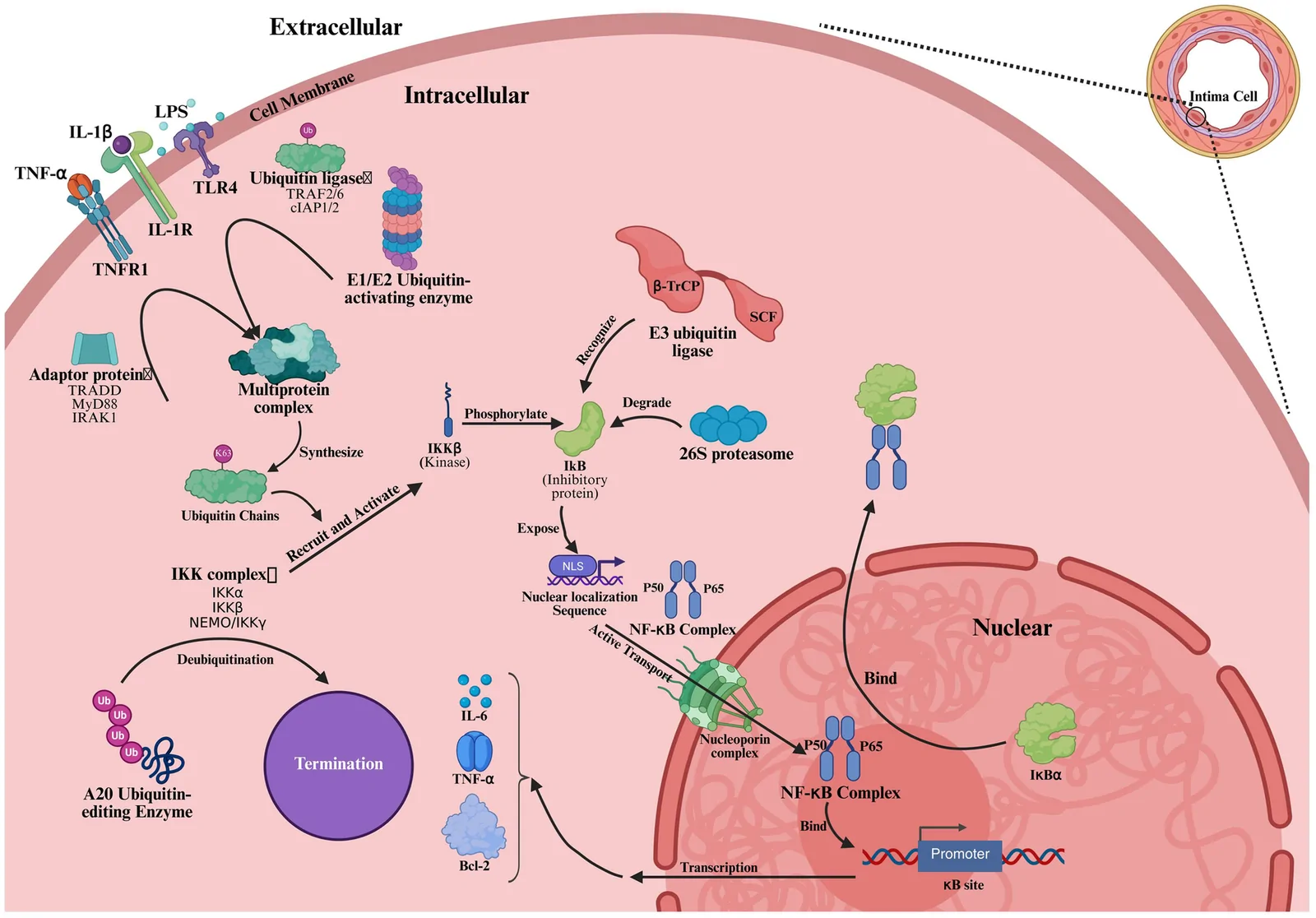
Canonical NF-κB activation in CAS. Extracellular stimuli (TNF-α, IL-1β, LPS) bind TNFR1/IL-1R/TLR4, recruiting adaptors (TRADD, MyD88, IRAK1) and ubiquitin ligases (TRAF2/6, cIAP1/2). K63-ubiquitin chains recruit the IKK complex comprising IKKα, IKKβ, and NEMO; IKKβ phosphorylates IκBα (Ser32/Ser36), leading to K48-ubiquitination and proteasomal degradation, nuclear translocation of p50/p65, and transcription of pro-inflammatory genes. Termination occurs via resynthesized IκBα and A20. per reviewer comment, the non-canonical antiviral kinases TBK1 and IKKi are not components of the canonical IKK complex and have been removed from the diagram. Direction of regulation and evidentiary status are indicated for each step. Figure created with BioRender (bio-render.com).

Multiple factors can induce activation of the NF-κB pathway, including cytokines, oxidative stress, and mechanical stress. Regarding oxidative stress, the flavonoid quercetin has been shown to suppress inflammatory responses in diabetic high-fat diet-induced CAS by modulating the AMPK/SIRT1/NF-κB pathway (29). Regarding mechanical stress, oscillatory shear stress (OSS) activates the TLR2-TAK1-IKK2-NF-κB pathway in endothelial cells, leading to inflammation and apoptosis, thereby promoting atherosclerotic plaque formation (30). Within established plaques, NF-κB activation is also associated with enhanced inflammatory activity of immune cells such as macrophages and T cells (31).

In summary, the NF-κB signaling pathway plays a central role in CAS-related inflammation, with its activation status directly correlating with plaque inflammation and stability. Modulating its activation through approaches such as quercetin or upstream TLR/TAK1-directed intervention may offer novel therapeutic strategies for CAS.

### 2.2 Regulatory roles of JAK/STAT and MAPK signaling pathways

The Janus kinase/signal transducer and activator of transcription (JAK/STAT) pathway primarily mediates cytokine signaling, thereby influencing immune cell activity and the intensity of inflammatory responses (32). These pathways play crucial roles in regulating cellular inflammation during CAS. For example, IL-27 modulates the expression of pro-inflammatory cytokines by activating the ERK signaling pathway (33); this evidence is drawn from a preterm-labor model and is cited here for pathway-level insight rather than as direct carotid-plaque data. Notably, the JAK/STAT pathway also cross-regulates with NF-κB and MAPK signaling, forming a complex inflammatory network (34–36). Studies confirm that IL-35 suppresses JAK/STAT signaling by modulating the LAIR1-PTPN11-JAK-STAT-FN1 network, thereby alleviating carotid artery inflammation in systemic lupus erythematosus (34); although reported in a lupus nephritis context, the JAK/STAT mechanism is conserved and relevant to carotid inflammation.

The mitogen-activated protein kinase (MAPK) pathway is activated in parallel with NF-κB from shared proximal nodes such as TAK1, with which it engages in bidirectional crosstalk, and includes three major subtypes: ERK, p38, and JNK, which extensively regulate cell proliferation, apoptosis, and inflammatory mediator expression (37). The ERK pathway primarily responds to growth factors (e.g., EGF, FGF) that bind to receptor tyrosine kinases (RTKs), leading to activation of the Ras-Raf-MEK-ERK cascade. Activated ERK dimers translocate to the nucleus and phosphorylate transcription factors such as Elk-1, c-Fos, and CREB (37, 38). The p38 pathway is predominantly activated by stress signals or pro-inflammatory cytokines (e.g., TNF-α, IL-1) via TAK1-MKK3/6, phosphorylating ATF-2 and other targets to drive IL-6 expression (39–41). The JNK pathway primarily governs apoptosis and cellular stress responses, phosphorylating c-Jun to promote pro-apoptotic gene expression (42, 43). These cascades are schematized in Figure 3, and a consolidated comparison of their substrates and pathological associations is provided in Table 1. (Figure 3 and Table 1).

**Figure 3:**
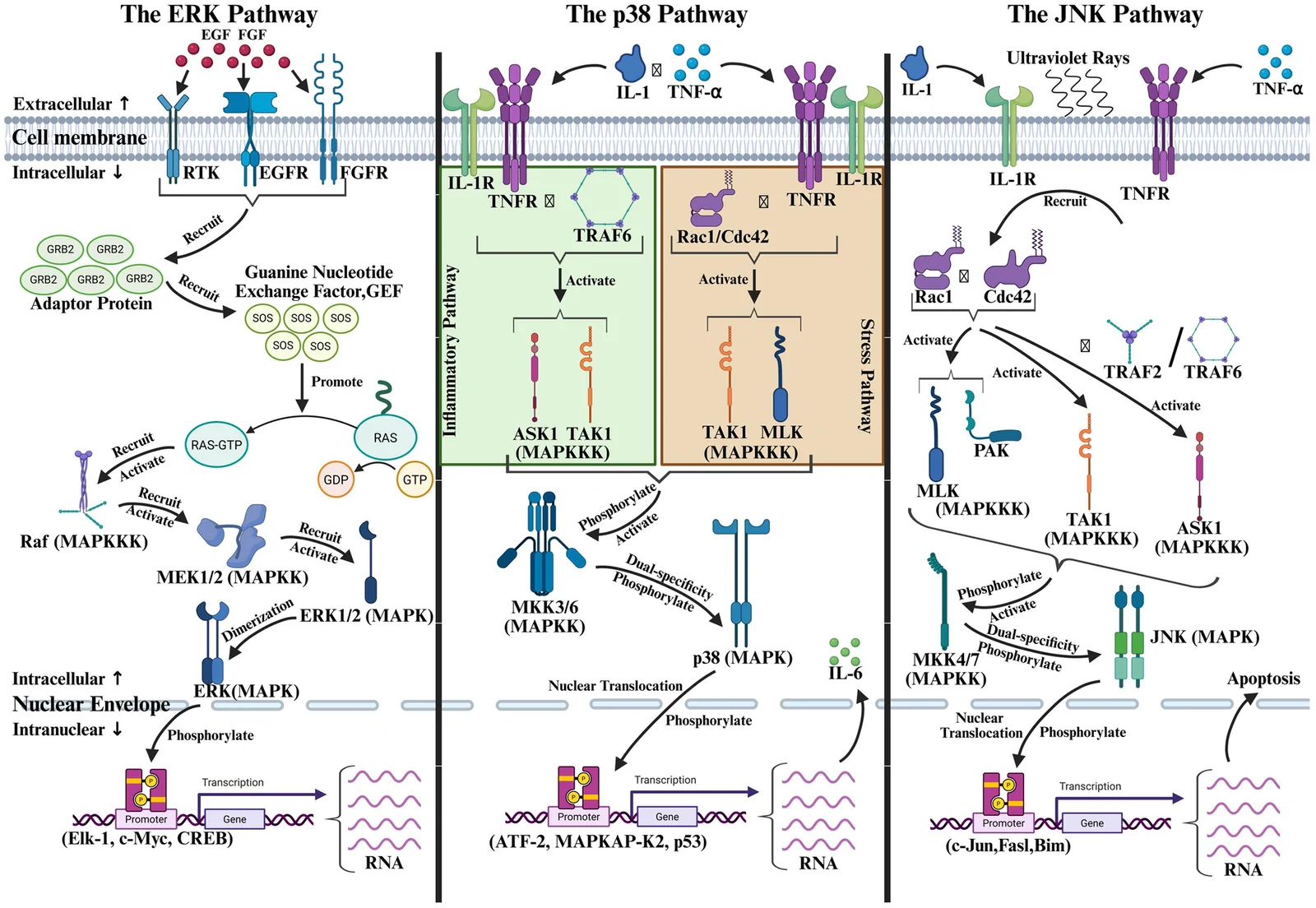
MAPK and JAK/STAT signaling in CAS. ERK, p38, and JNK are activated in parallel with NF-κB from shared proximal nodes (e.g., TAK1) and engage in bidirectional crosstalk. Substrates shown are consistent with the main text: ERK phosphorylates Elk-1, c-Fos, and CREB; p38 phosphorylates ATF-2; JNK phosphorylates c-Jun. The JAK/STAT axis mediates cytokine signaling and cross-regulates NF-κB/MAPK. Arrows indicate activation. Figure created with BioRender (bio-render.com).

**Table 1:** Comparison of the three major MAPK cascades in atherosclerotic inflammation (converted from the former Figure 4).

Pathway	Major upstream activators	Key substrates/TFs	Primary functional output	Pathological association
ERK	Ras-Raf-MEK (growth factors, RTKs)	Elk-1, c-Fos, CREB	Proliferation; inflammatory gene expression	Plaque growth
p38	TAK1-MKK3/6 (stress, TNF-alpha, IL-1)	ATF-2, p53	IL-6 and inflammatory mediator expression	Plaque inflammation
JNK	ASK1-MKK4/7 (Rac1, Cdc42)	c-Jun	Apoptosis; pro-apoptotic genes (FasL, Bim)	Foam-cell death; fibrous-cap thinning

Research indicates that quercetin, the primary active component in the Huoxuehuayu Tongluo formula, inhibits both MAPK and JAK/STAT signaling pathways, thereby reducing the expression of TNF-α, IL-1β, and IL-6 and alleviating plaque inflammation (44, 45). In osteoarthritis models, STEAP3 has been shown to activate MAPK and JAK/STAT pathways by inhibiting Rab7A activity and promoting RACK1 activity, thereby promoting inflammatory responses and disease progression (46); this evidence is cited for its mechanistic insight into MAPK/JAK-STAT regulation and is not derived from carotid plaque. In summary, the MAPK and JAK/STAT signaling pathways play pivotal roles in regulating CAS-associated inflammation, and targeting these pathways offers promising strategies for therapeutic intervention.

### 2.3 Types of inflammatory mediators and their expression characteristics

CAS involves an inflammatory process coordinated by multiple mediators. Cytokines such as tumor necrosis factor-α (TNF-α), interleukin-1β (IL-1β), and interleukin-6 (IL-6) are highly expressed at lesion sites (47). Significantly elevated plasma IL-6 levels have been observed in patients with progressive supranuclear palsy, positively correlating with CAS severity, suggesting that IL-6 plays a crucial role in inflammation-mediated plaque formation (48). Chemokines such as monocyte chemotactic protein-1 (MCP-1/CCL2) regulate the recruitment of immune cells to atherosclerotic lesions, promoting local inflammatory cell infiltration and foam cell formation (47, 49). Reactive oxygen species (ROS) and oxidative stress products are abundantly generated in the inflammatory environment, inducing cellular damage and activating inflammatory signaling pathways, thereby exacerbating endothelial dysfunction and plaque instability (48, 50). Matrix metalloproteinases (MMPs) play a central role in plaque matrix degradation, regulating fibrous cap stability; their overexpression increases the risk of plaque rupture (51). Furthermore, activation of the alternative pathway of the complement system enhances local inflammatory responses, with components such as C3, CFB, and CFH closely associated with carotid plaque formation (52). Collectively, the inflammatory mediators in CAS are characterized by synergistic overexpression of multiple pro-inflammatory factors and accumulation of ROS, creating a complex inflammatory microenvironment that drives plaque formation and progression.

### 2.4 The role of inflammatory mediators in plaque formation and progression

Inflammatory mediators contribute to plaque formation and progression through multiple mechanisms. Pro-inflammatory factors such as TNF-α, IL-1β, and IL-6 induce endothelial dysfunction, leading to reduced nitric oxide synthesis and increased vascular permeability, thereby promoting deposition of low-density lipoprotein (LDL) and its oxidized products in the vascular endothelium (52, 53). These mediators also induce vascular smooth muscle cells (VSMCs) to switch from a contractile to a migratory-proliferative phenotype, promoting their migration into the intima and contributing to fibrous cap formation (54); however, excessive activation leads to matrix degradation and instability (54, 55). Sustained inflammation triggers massive release of MMPs, accelerating fibrous cap degradation and increasing the risk of plaque rupture (51). ROS and oxidative stress products not only directly damage cells but also activate multiple inflammatory signaling pathways, exacerbating plaque inflammatory burden and instability (56). Complement system activation contributes to plaque development by enhancing inflammatory cell infiltration and pro-inflammatory mediator release. Additionally, inflammation in obesity-associated adipose tissue indirectly promotes plaque formation by secreting cytokines and chemokines—including TNF-α, CXCL2, and CCL2—that enhance monocyte chemotaxis (57–59). In summary, inflammatory mediators promote plaque formation, maturation, and instability through multiple pathways, establishing themselves as critical drivers of CAS pathology (60, 61) (Table 2).

**Table 2:** Key studies on chemokine axes and inflammatory mediators in carotid atherosclerosis.

First author	Year	Study design	Model/sample	Key finding	Clinical implication
Segers D	2011	Experimental + human	ApoE−/− mice; human plaques	CXCL10 elevation in carotid plaques correlates with vulnerable phenotype; neutralization stabilizes plaques *in vivo*.	CXCL10 blockade as a plaque-stabilizing strategy.
Weber C	2011	Experimental (mouse)	Murine atherosclerosis	CCL17-expressing dendritic cells accumulate in advanced lesions; CCL17 deficiency attenuates atherogenesis.	CCL17/DC axis as an immunomodulatory target.
Akhtar S	2013	Experimental (mouse)	ApoE−/− mice	CXCL12 promotes plaque stabilization and progenitor cell recruitment without increasing lesion size.	Pro-stabilizing chemokine; caution against broad CXCL12 blockade.
Doring Y	2017	Experimental + human	Mouse and human arteries	Vascular CXCR4 preserves endothelial barrier function and SMC contractile phenotype, limiting atherosclerosis.	CXCR4 integrity as a protective axis.
Georgakis MK	2019	Mendelian randomization	GWAS summary statistics	Genetically determined circulating cytokine levels associate with ischemic stroke risk.	Genetically supported cytokine targets for stroke prevention.
Georgakis MK	2021	Human observational	Human atherosclerotic lesions	CCL2 levels correlate with plaque vulnerability and are higher in symptomatic vs. asymptomatic lesions.	CCL2 as a vulnerability marker; symptomatic-plaque stratification.
Georgakis MK	2022	Mechanistic review	Preclinical/human evidence	The CCL2/CCR2 axis orchestrates monocyte trafficking from bone marrow to plaques.	CCR2 antagonism as anti-recruitment therapy.
Velasquez IM	2023	Mendelian randomization	GWAS + cIMT	Plasma CXCL8 (IL-8) causally affects carotid intima-media thickness.	IL-8 targeting for subclinical disease.
Prapiadou S	2024	Proteogenomic integration/MR	Human proteomics + genetics	Proteogenomic integration identifies CXCL10 as a causal mediator of IL-6-driven atherosclerosis.	CXCL10 as a genetically supported therapeutic target.
Georgakis MK	2025	Genetic association	Human sequencing	Rare damaging CCR2 variants lower lifetime cardiovascular risk but increase infection susceptibility.	CCR2 targeting entails an infection trade-off.
Lombardi M	2025	Cross-sectional (human)	Carotid plaque vs. AAA patients	P2X7, GM-CSF, IL-1beta, CXCL1 fingerprint distinguishes carotid plaque from AAA.	P2X7 as a diagnostic/therapeutic target.
Sun JX	2025	Cross-sectional screening	9,503 Chinese adults	AISI outperforms SII/SIRI for carotid plaque prediction.	AISI for population risk stratification.
Beyaz MO	2026	Prospective (human)	CEA patients, pre/post-op	CEA reduces ten inflammatory biomarkers (IL-6 −29.3%, NLR −28.6%), with greater effect in symptomatic/unstable plaques.	CEA inflammatory benefit; perioperative biomarker monitoring.

To refocus this review on the carotid vascular bed specifically, we emphasize several structural and cellular correlates of plaque instability that are particularly tractable in the carotid because endarterectomy makes human plaque tissue routinely available for immunological study (2, 62). These include intraplaque hemorrhage, necrotic core expansion, adventitial neovascularization, and defective efferocytosis of apoptotic cells (2). Intraplaque hemorrhage drives erythrocyte-derived cholesterol loading and macrophage infiltration; expanded necrotic cores reflect failed efferocytosis and amplify NLRP3 inflammasome activation (63, 64); and adventitial neovascularization sustains leukocyte trafficking into the lesion (2). Comparisons of symptomatic vs. asymptomatic carotid plaques consistently show that symptomatic lesions harbor more macrophage inflammation, more pronounced T cell activation, and higher levels of CCL2 and related chemokines (65–67), providing a human, carotid-specific rationale for the inflammatory mechanisms discussed below.

### 2.5 Mechanism of action of the NLRP3 inflammasome

The NLRP3 inflammasome functions as an intracellular danger signal sensor, detecting various stimuli including lipid metabolism disorders, oxidative stress, and cellular damage (63, 68). Its activation promotes the maturation and secretion of pro-inflammatory cytokines IL-1β and IL-18, thereby enhancing local inflammatory responses and accelerating CAS progression (63, 69).

In atherosclerotic models, NLRP3 inflammasome activation is regulated by oxidative-stress and metabolic cues—including mitochondrial ROS, mtDNA release, and K^+^ efflux—and can be mitigated by Nrf2-activating agents such as melatonin, which suppress NLRP3-driven vascular inflammation (70–72). Furthermore, in smoking-exposed human aortic endothelial cells, tobacco smoke extract-induced ROS generation activates the NLRP3 inflammasome via the Nrf2-ROS-NLRP3 axis. Conversely, melatonin suppresses NLRP3 activation by activating the Nrf2 pathway, thereby mitigating smoking-induced vascular injury and plaque formation (73, 74). These findings suggest that the NLRP3 inflammasome is a critical node regulating plaque inflammation and that its activation can be modulated by antioxidant signaling pathways (63).

In advanced carotid atherosclerotic plaques, pyroptosis-related genes such as GSDMD, CASP1, NLRC4, AIM2, and IL-18 are upregulated, indicating that enhanced pyroptosis contributes to plaque formation and rupture (75, 76). Activation mechanisms of the NLRP3 inflammasome involve signals such as mitochondrial DNA release and K^+^ efflux, providing potential targets for therapeutic intervention (71, 72).

In summary, the NLRP3 inflammasome perceives danger signals, promotes IL-1β and IL-18 secretion, and drives pyroptosis, thereby contributing to the inflammatory progression and instability of carotid atherosclerotic plaques. Precise intervention targeting this inflammasome holds promise as a novel therapeutic strategy for CAS.

### 2.6 Other inflammasomes

Beyond NLRP3, several other inflammasomes—including NLRP1, NLRP6, NLRP12, NLRC4, and AIM2—have been characterized in other tissues and disease contexts, but their direct roles in CAS pathogenesis remain poorly defined (75, 77–79). Of these, NLRC4 and AIM2 are of particular interest because, together with NLRP3, they encode plaque pyroptosis effector genes that are upregulated in advanced carotid lesions (75); however, whether they function as active inflammasome sensors within carotid plaques, as opposed to bystander transcripts, awaits direct experimental confirmation. We have therefore removed the off-target tissue-specific descriptions (e.g., NLRP1 in keratinocytes, NLRP6 in goblet cells) from the main text and flagged these sensors as unconfirmed in CAS in Figure 1.

### 2.7 Involvement of the monocyte-macrophage lineage

Monocyte-macrophage lineage cells play a central role in the inflammatory process of CAS. Monocytes are recruited to lesion sites by inflammatory signals and differentiate into macrophages within the local microenvironment (80). These macrophages form foam cells by phagocytosing oxidized low-density lipoprotein (ox-LDL), representing one of the key cell types in atherosclerotic plaque formation (53, 81). Beyond accumulation within plaques, macrophages promote local inflammation and matrix degradation by secreting multiple pro-inflammatory factors (e.g., TNF-α, IL-1β, IL-6) and MMPs, leading to plaque instability (67, 82, 83).

The polarization state of macrophages exerts crucial regulatory effects on disease progression. M1 macrophages exhibit a pro-inflammatory phenotype, secreting abundant inflammatory mediators that amplify inflammatory responses and promote plaque progression and rupture. In contrast, M2 macrophages possess anti-inflammatory and reparative functions, aiding in inflammation resolution and tissue repair (84). The M1/M2 ratio shifts dynamically across stages of CAS: early stages are characterized by M1-dominant polarization driving inflammation and lesion expansion, whereas later stages show partial conversion to M2-type in an attempt to stabilize plaques (85). Research further indicates that macrophages influence their inflammatory state and cellular senescence through epigenetic mechanisms, such as RNA N6-methyladenosine (m6A) modification, thereby affecting disease progression (86). Additionally, transcription factors like IRF5 promote the maintenance of pro-inflammatory phenotypes in macrophages, intensifying plaque inflammation (87). Thus, the monocyte-macrophage lineage not only mediates inflammatory responses but also serves as a key determinant of plaque formation and stability (66), making it a critical therapeutic target (88) (Figure 4).

**Figure 4:**
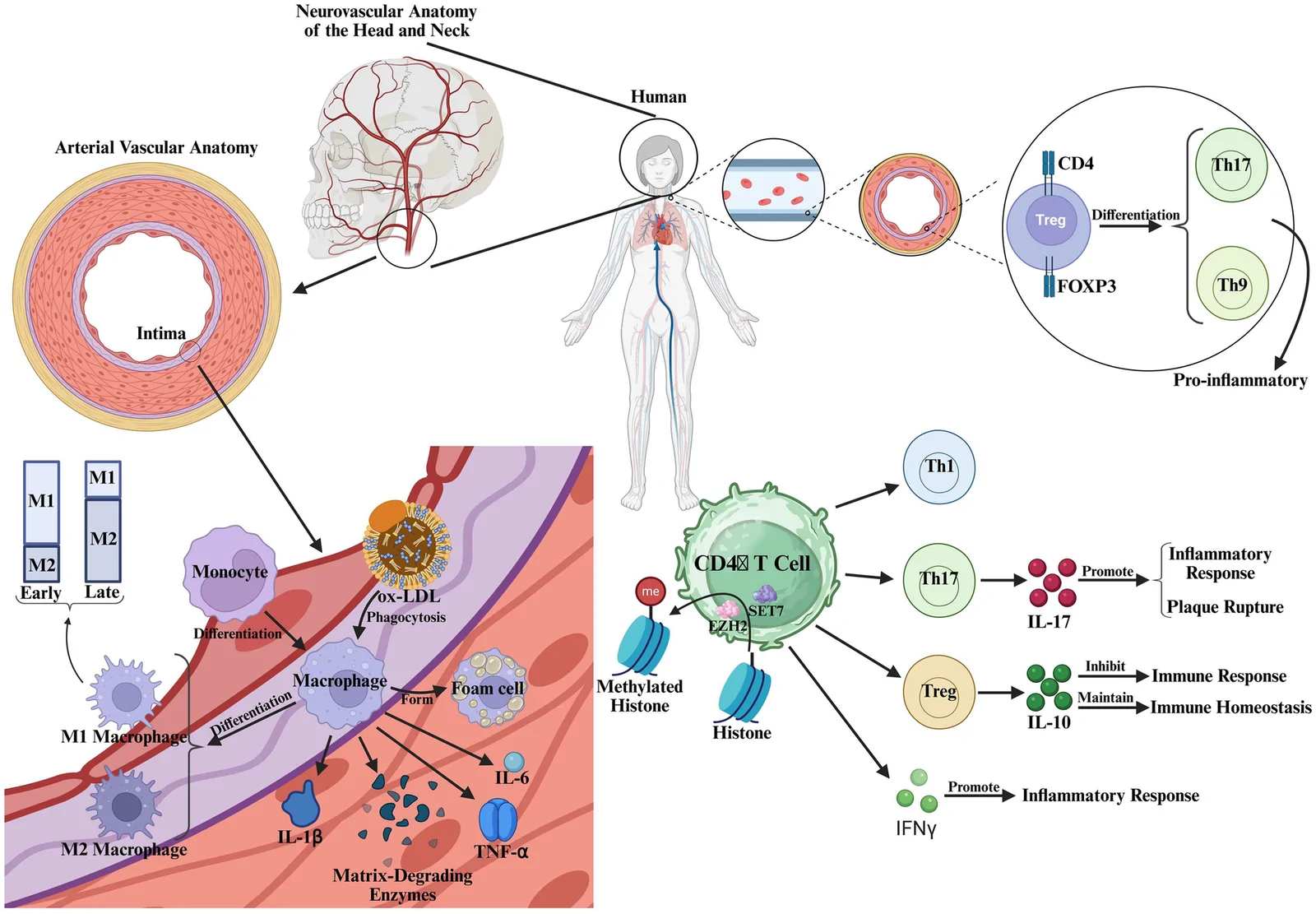
Immune cell populations and their functional balance in carotid plaque stability. Monocyte-derived macrophages (M1/M2, with TREM2+ and other heterogeneous states), Th1/Th17/Treg subsets, CD8+ T cells including the SLC4A10+ population (protective, diminishing IFN-γ/GZMB), dendritic cells, neutrophils/NETs, and tertiary lymphoid organs are depicted in relation to plaque instability versus stability. The figure summarizes Sections 3.7–3.10. Figure created with BioRender (bio-render.com).

Importantly, the classical M1/M2 dichotomy is increasingly recognized as an oversimplification. Single-cell and spatial transcriptomic studies of human atherosclerotic plaques have revealed substantial macrophage heterogeneity, including TREM2-expressing macrophages, lipid-associated macrophages, interferon-responsive macrophages, inflammatory macrophages, and resident-like macrophage populations, whose functions extend beyond a simple pro-/anti-inflammator*y* axis (89–91). TREM2+ macrophages in particular link lipid sensing, efferocytosis, and metabolic reprogramming to plaque behavior: they can contribute to lipid accumulation and inflammation in early lesions while participating in plaque stabilization and tissue repair in later phases (92, 93). Accordingly, the M1/M2 framework is retained in this review as a useful heuristic for conceptualizing net inflammatory balance, but it should be understood as a simplification of a much richer, dynamically shifting macrophage state space that single-cell data are now defining (85, 89).

### 2.8 The role of T cells and their subpopulations

T cells and their subsets play complex regulatory roles in CAS-associated inflammation (94). CD4^+^ T cells, as primary effector cells of the adaptive immune system, secrete multiple pro-inflammatory cytokines such as interferon-γ (IFN-γ), thereby amplifying local inflammatory responses and exacerbating plaque instability (67, 95). Multiple activated CD4^+^ T cell subsets have been identified within plaques, including pro-inflammatory Th1 and Th17 cells. Th17 cells promote inflammation and plaque rupture by secreting IL-17 (67). Conversely, regulatory T cells (Tregs) suppress immune responses by secreting anti-inflammatory factors like IL-10, maintaining immune homeostasis and preventing excessive inflammation. This protective effect contributes to plaque stability (95–97). In clinical samples, reduced Treg numbers correlate with plaque vulnerability (67). Studies also indicate that epigenetic regulation of T cells, such as histone methylation mediated by EZH2, modulates T cell polarization and function, influencing plaque inflammation (6, 7). The activation status and subset composition of T cells correlate with clinical symptoms; for instance, an increased proportion of activated T cells in plaques of symptomatic patients is associated with plaque vulnerability (98). Furthermore, CD4^+^FOXP3^+^ Treg cells in symptomatic patients may transdifferentiate into pro-inflammatory Th17 and Th9 cells, exacerbating plaque inflammation (98, 99). Collectively, the balance among T cell subsets and their secreted cytokines jointly determines plaque inflammation and stability, establishing T cells as critical targets for immunotherapy in CAS (95, 100, 101) (Figure 4).

Beyond the classical Th1/Th17/Treg classification, single-cell T cell receptor profiling and transcriptomic atlases have uncovered considerable functional diversity, including exhausted and tissue-resident T cell states, breakdown of T cell tolerance checkpoints, and clonal expansion within plaques (85, 101, 102). These findings indicate that T cell behavior in CAS is shaped by local antigen-driven activation and chronic stimulation rather than by subset identity alone.

Recent studies have confirmed that SLC4A10+ CD8^+^ T cells are significantly enriched in carotid atherosclerotic plaques; mechanistic studies indicate that SLC4A10 impedes atherosclerosis by diminishing IFN-γ and GZMB levels in CD8^+^ T cells via the MAPK pathway, i.e., SLC4A10 marks a T cell population with a protective rather than a destabilizing role (103). Concurrently, CIITA-mediated antigen presentation in macrophages is activated, effectively driving local T-cell inflammatory responses and accelerating plaque progression (89, 104). More importantly, the discovery of tertiary lymphoid organ-like structures (TLOs) in atherosclerotic plaques has opened new perspectives for understanding the mechanisms of local adaptive immune responses (105). TLOs are composed of fibroblast-like smooth muscle cells that recruit B cells and T cells by secreting chemokines such as CXCL12, thereby promoting local inflammatory responses (106, 107). Clinical cohort studies indicate that plaques containing TLOs are associated with cerebrovascular events and may represent a structural biomarker of plaque vulnerability and a candidate therapeutic target; the magnitude of this association and its independence from conventional risk factors require prospective validation in dedicated carotid cohorts (105).

### 2.9 Contributions of other immune cells

Beyond monocytes/macrophages and T cells, other immune cells also play important roles in CAS. Dendritic cells (DCs), as professional antigen-presenting cells, can activate adaptive immune responses and promote T cell activation and differentiation. Different DC subtypes exhibit distinct distributions and functions within plaques, participating in the regulation of plaque inflammation (89, 108). Neutrophils, key components of innate immunity, enhance local inflammatory responses by releasing neutrophil extracellular traps (NETs), promote thrombosis, and increase the risk of plaque rupture. NET formation is closely associated with plaque progression, and neutrophil activation correlates with plaque instability (89). Natural killer (NK) cells participate in immune surveillance and regulation of inflammation; their role in CAS remains incompletely understood, though studies suggest they may modulate the atherosclerotic immune microenvironment (89). Additionally, *γδ* T cells have been implicated in plaque inflammation and may contribute to pathological processes through pro-inflammatory cytokine secretion (89). In summary, the inflammatory microenvironment of CAS involves the coordinated action of multiple immune cell populations, whose interactions and functional states collectively determine the degree of inflammation and disease progression, providing a rich array of targets for immune-directed therapies (89).

### 2.10 Single-cell, spatial, and multi-omics insights into carotid plaque immunity

Recent advances in carotid atherosclerosis research have been driven largely by single-cell RNA sequencing (scRNA-seq), spatial transcriptomics, spatial proteomics, and multi-omics integration. These technologies have substantially refined our understanding of immune-cell heterogeneity, endothelial dysfunction, and cell-cell communication within carotid plaques. Single-cell transcriptomics of human carotid plaques has resolved the plaque immune landscape into functionally distinct myeloid and lymphoid states, including the TREM2+ macrophage and SLC4A10+ CD8+ T cell populations discussed above, and has revealed crucial cell subsets associated with cerebrovascular events (89). Cross-species scRNA-seq comparing human carotid and experimental murine atherosclerosis has uncovered divergent T cell activation phenotypes, cautioning against direct translation from animal models (85). Spatial transcriptomics has further localized tertiary lymphoid organ-like structures within plaques and mapped their cellular neighborhoods (105), while integration of CFD hemodynamics with CyTOF immunophenotyping has linked disturbed flow fields to specific immune microenvironments in the carotid bifurcation (109). At the multi-omics level, proteogenomic integration has identified CXCL10 as a downstream causal mediator of IL-6-driven atherosclerosis, providing genetically supported therapeutic targets (58, 110), and plasma soluble TREM2 has been correlated with carotid atherosclerotic burden (92). Together, these approaches are transforming CAS from a pathway-centric paradigm into a spatially resolved, cell-state-resolved system, and they underpin several of the macrophage and T cell findings integrated into Sections 3.7 and 3.8.

### 2.11 Non-coding RNA, epigenetic, and hemodynamic regulation of carotid inflammation

To deliver on the advances highlighted in the Introduction, we briefly synthesize three regulatory layers that are increasingly implicated in CAS. Non-coding RNAs regulate inflammatory gene expression and macrophage phenotype in carotid lesions: miR-223 exerts translational control of proatherogenic genes in macrophages (9), the lncRNA Lnc_000048 reduces plaque stability by recruiting KDM1A and promoting H3K4 methylation of MAP2K2 (10), and miRNAs act as sentinels of carotid artery thromboembolism (11). Epigenetic mechanisms—including EZH2-mediated histone modification that shapes T cell plasticity (6, 7) and RNA m6A modification that modulates macrophage inflammatory state and aging-related programs (86, 94)—provide durable, druggable control of inflammatory outputs. Finally, hemodynamic forces are a defining feature of the carotid bifurcation: stable laminar shear stress induces anti-inflammatory programs such as KLK10 (13) and endothelial galectins (12), whereas oscillatory shear stress activates TLR2-TAK1-IKK2-NF-κB signaling and promotes inflammation (30); computational fluid dynamics combined with immune profiling has begun to map these flow niches onto local immune cell composition in CAS (109).

## 3 Discussion

As the core pathological basis of cerebrovascular events, carotid atherosclerosis (CAS) is fundamentally an inflammatory disease (111). While decades of research have established inflammation as a central driver of plaque formation, progression, and rupture, the precise molecular and cellular networks governing this process continue to be refined. In this review, we have comprehensively synthesized current knowledge regarding the signaling pathways, inflammatory mediators, and immune cell populations that orchestrate CAS-associated inflammation. Here, we discuss the key insights emerging from this synthesis, identify persistent knowledge gaps, and propose future directions for translating mechanistic understanding into clinical benefit.

### 3.1 Integration of inflammatory signaling networks

At the molecular level, the NF-κB, JAK/STAT, and MAPK signaling pathways, together with the NLRP3 inflammasome, constitute the core hubs of the inflammatory response in CAS (68, 112, 113). Our synthesis reveals that these pathways do not operate in isolation but rather form an integrated and highly interconnected network. For instance, MAPK cascades and NF-κB are activated in parallel from shared proximal nodes such as TAK1 and engage in bidirectional crosstalk, creating feed-forward loops that amplify and sustain inflammation (113). The NLRP3 inflammasome, activated by lipid crystals, oxidative stress, and mitochondrial damage, serves as a critical node linking metabolic danger signals to IL-1β/IL-18-driven inflammation (68).

However, the clinical translation of these pathway-centric discoveries has proven challenging. The remarkable redundancy and crosstalk among signaling modules means that targeting a single molecule may be insufficient to suppress pathogenic inflammation, as compensatory mechanisms can rapidly restore signaling output (35, 36). Conversely, broad inhibition of master regulators such as NF-κB risks unacceptable on-target toxicity due to their essential roles in host defense and tissue homeostasis (25). Thus, future therapeutic strategies may benefit from targeting upstream initiators (e.g., specific TLRs), downstream effectors with restricted expression (e.g., NLRP3), or cell-type-specific delivery to achieve precision immunomodulation (114).

### 3.2 The dual role of inflammatory mediators and the therapeutic window

Inflammatory mediators, including cytokines, chemokines, ROS, and MMPs, play indispensable roles in regulating immune cell recruitment and activation. Our analysis highlights a key dichotomy: these molecules serve both as essential components of host defense and as catalysts of pathological progression (54, 55, 63). For example, while MCP-1/CCL2 is required for monocyte recruitment to sites of injury, its sustained overproduction drives excessive macrophage accumulation and foam cell formation, accelerating plaque growth (47, 56). Similarly, MMPs are necessary for tissue remodeling and repair, but their dysregulated expression degrades the fibrous cap, precipitating plaque rupture (115, 116).

This dual nature imposes important constraints on therapeutic development. Complete neutralization of a given mediator may interfere with protective homeostatic functions, whereas partial modulation or targeting of downstream receptor-effector coupling may offer a safer window. The success of the CANTOS trial—targeting IL-1β with canakinumab—demonstrates that pathway-specific anti-inflammatory strategies can reduce cardiovascular events, albeit with an increased risk of infection (14, 117). Equally informative are the negative and partially positive trials: in CIRT, low-dose methotrexate lowered neither inflammatory markers (IL-1β, IL-6, CRP) nor cardiovascular events (118), underscoring that not all anti-inflammatory approaches translate. By contrast, colchicine—the first anti-inflammatory agent to reach regulatory approval for atherosclerotic cardiovascular disease—reduced ischemic events in both the COLCOT (119) and LoDoCo2 (120) trials, although an excess of pneumonia was observed in COLCOT. Together, these trials show that the benefit of anti-inflammatory therapy is class- and target-specific, and that systemic inhibition carries a real infection penalty. We therefore emphasize that the M1/M2 framework remains a useful heuristic for conceptualizing net inflammatory balance, whereas the single-cell data summarized in Sections 3.7 and 3.10 better capture the macrophage state space that should guide next-generation, cell-type-targeted interventions. These findings argue for careful risk-benefit assessment and for intermittent or lesion-targeted approaches over chronic systemic inhibition.

### 3.3 Immune cell plasticity and plaque stability

Among immune cells, the monocyte-macrophage lineage and T cell subsets emerge as key determinants of plaque fate. Our synthesis emphasizes that it is not merely the presence but the functional polarization state of these cells that governs disease outcomes. M1 macrophages and Th1/Th17 cells promote inflammation and plaque vulnerability, while M2 macrophages and Tregs exert anti-inflammatory and stabilizing effects (85, 88). The dynamic shift in M1/M2 and Treg/Th17 ratios across disease stages suggests that rebalancing, rather than eliminating, immune responses may be a viable therapeutic strategy.

Emerging evidence further implicates additional immune populations, including dendritic cells, neutrophils, and even tertiary lymphoid organs within human plaques, in modulating local inflammation (89, 109, 121). Furthermore, SLC4A10+ CD8+ T cells and macrophage CIITA-mediated antigen presentation shape plaque immunity, highlighting the complexity of the immune microenvironment; the discovery of tertiary lymphoid organs in carotid plaques also opens new avenues for understanding how local adaptive immune responses are organized and maintained (105). These findings collectively argue for a multi-cell-type, multi-target approach to immunomodulation in CAS.

### 3.4 From bench to bedside: opportunities and challenges

Significant progress has been made, yet several critical gaps remain. First, most mechanistic insights derive from animal models that incompletely recapitulate human disease, as highlighted by cross-species single-cell transcriptomic comparisons revealing divergent T cell activation states (85). Second, the spatiotemporal dynamics of inflammatory cells within evolving human plaques remain poorly defined; most human studies provide only static snapshots from endarterectomy specimens. Third, the translation of biomarker findings into clinical practice has been slow, with few inflammatory markers incorporated into routine risk stratification algorithms.

Looking forward, several priorities should guide future research. Advanced imaging techniques (e.g., molecular MRI, PET tracers for specific inflammatory targets) could enable non-invasive monitoring of plaque inflammation in living patients. Single-cell multi-omics and spatial transcriptomics applied to human plaque samples will resolve cellular heterogeneity and intercellular communication networks with unprecedented resolution (89, 101, 105). Nanoparticle-based drug delivery systems offer the potential for lesion-specific targeting of anti-inflammatory agents, minimizing systemic exposure. Finally, Mendelian randomization and proteogenomic studies such as those identifying CXCL10 as a causal mediator of IL-6-driven atherosclerosis (110) provide a roadmap for validating therapeutic targets with genetic evidence. Clinically, circulating inflammatory indices (e.g., NLR, SII, SIRI, AISI) and CEA-induced biomarker dynamics are already being evaluated for carotid plaque risk stratification (62, 122), while soluble TREM2 has emerged as a candidate plasma biomarker of macrophage activation in carotid atherosclerosis (92); molecular imaging and these emerging biomarkers together represent the most tractable near-term route to precision risk assessment in CAS.

### 3.5 Concluding remarks

In summary, the inflammatory mechanisms underlying CAS involve a highly integrated, multi-level network of signaling pathways, mediators, and immune cells, now being resolved at single-cell and spatial resolution. Multidisciplinary collaboration integrating molecular biology, immunology, imaging, and clinical medicine will be essential to propel deeper mechanistic exploration and facilitate effective translation from bench to bedside. By navigating the delicate balance between suppressing pathogenic inflammation and preserving protective immunity, we can aspire to stabilize high-risk carotid plaques, reduce the incidence of cerebrovascular events, and ultimately improve patient outcomes.

## 4 Conclusion

Carotid atherosclerosis (CAS) is fundamentally driven by inflammation. In this review, we have comprehensively delineated the hierarchical organization of NF-κB, MAPK, JAK/STAT, and NLRP3 inflammasome signaling, the functional diversity of inflammatory mediators, and the coordinated actions of immune cells within the plaque microenvironment. Three key principles emerge: (i) core signaling pathways form an integrated network that perpetuates pro-inflammatory signaling; (ii) the balance between M1/M2 macrophages and Treg/Th17 cells determines plaque stability, a balance now refined by single-cell macrophage and T cell heterogeneity data; and (iii) clinical translation of these insights remains hindered by pathway redundancy and lack of spatial specificity. Future priorities should include deciphering the spatiotemporal dynamics of plaque inflammation using single-cell multi-omics, developing targeted immunomodulatory strategies (e.g., nanoparticle delivery, gene editing), and integrating inflammatory biomarkers into personalized risk assessment. By translating mechanistic discoveries into clinical practice, we can aspire to stabilize high-risk plaques and reduce the burden of ischemic stroke.
